# Tai Chi exercise for sleep problems in older adults: A protocol of systematic review and meta-analysis

**DOI:** 10.1097/MD.0000000000017556

**Published:** 2019-11-11

**Authors:** Yuhao Si, Cenyi Wang, Jinghui Zheng, Yang Guo, Guihua Xu, Yong Ma

**Affiliations:** a The First School of Clinical Medicine, Laboratory for New Techniques of Restoration & Reconstruction of Orthopedics and Traumatology, Nanjing University of Chinese Medicine, Nanjing; b The First Affiliated Hospital of Guangxi University of Chinese Medicine, Nanning; c Rangos School of Health Sciences, Duquesne University, Pittsburgh; d Department of Cardiology, Ruikang Hospital Affiliated to Guangxi University of Chinese Medicine, Nanning; e School of Nursing, Nanjing University of Chinese Medicine; f Department of Traumatology & Orthopedics, Affiliated Hospital of Nanjing University of Chinese Medicine; g College of Basic Medicine, Nanjing University of Chinese Medicine, Nanjing, China.

**Keywords:** older adults, protocol, sleep problems, sleep quality, systematic review, Tai Chi

## Abstract

**Background:**

Sleep plays a crucial role in the general health of the human body. However, sleep problems become increasingly severe with age, and its incidence is on the rise. Notably, Tai Chi exercise may be an efficacious means to ameliorate sleep problems among older adults. This is especially since Tai Chi has been widely used to manage sleep problems in China for many years, but there is limited evidence regarding its effectiveness. We will conduct this study aiming to investigate the efficacy of Tai Chi as a complementary and alternative therapy for sleep problems in older adults.

**Methods:**

We will systematically search Scopus, Embase, PubMed, Cochrane Library, China National Knowledge Infrastructure, and Wanfang database from their inceptions to August 2019. Only randomized controlled trials of Tai Chi for sleep problems among older adults will be considered for inclusion and the Pittsburgh sleep quality index will be the primary outcome measurement. Moreover, study selection, data extraction, and the evaluation of the methodological quality of trials will each be independently completed by at least 2 researchers. We will employ the software Stata version 12.0 to implement the statistical analysis.

**Results:**

The current systematic review and meta-analysis will provide synthesized results of the effectiveness and safety of Tai Chi exercise for sleep problems among older adults.

**Conclusion:**

This systematic review and meta-analysis will provide high-quality evidence on the effectiveness and safety of Tai Chi exercise for sleep problems in older adults.

**Registration:**

PEROSPERO CRD42019129782.

## Abstract 2

Supplemental Digital Content is available in the text

## 1 Introduction

Sleep problems, especially insomnia, that occurs independently or results from other comorbidities, constitute one of the most common health care problems in older adults.^[1]^ Notably, 58% of people suffer from sleep difficulties at least several times per week.^[2]^ It is recognized that sleep has a significant impact on human health condition and sleep disorders can contribute to increased fatigue, emotional or psychiatric disturbance, declined cognitive function, and poor quality of life; this is especially true among the elderly population.^[3–5]^ However, approximately 85% of insomniacs (including people under the threshold of insomnia diagnosis but with sleep complaints) tend to not seek professional consultation and intervention.^[6]^ Unfortunately, pharmacological therapy remains a universal method in managing chronic sleep problems among those who use sleep-promoting treatments.^[7,8]^ This method can contribute to numerous adverse effects such as drug dependence, fatigue, residual daytime sedation, headaches, and hallucinations.^[9]^

Cognitive behavior therapy (CBT) is highly recommended as the first-line choice in alleviating insomnia by both the American College of Physicians and the European Sleep Research Society.^[10,11]^ CBT, which usually encompasses sleep restriction, sleep hygiene, cognitive therapy, relaxation, and reduction of stimulus, is considered more efficacious than pharmacotherapy on sleep quality enhancements,^[12,13]^ and with extremely mild side effects. Nonetheless, the implementation of CBT may lack feasibility in most grassroots healthcare institutions. It is also a low cost-efficient among people with a moderate level of sleep complaints rather than people with diagnostic insomnia. This is largely attributed to the fact that highly trained therapists are always involved in the initiation and administration of CBT.^[12]^

Tai Chi is a traditional Chinese martial art encompassing slow physical movements combined with concentration and meditation.^[14]^ The integrated form of body, breath, and mind in Tai Chi exercise which aims to achieve greater awareness and a sense of well-being has been proven effective in improving health outcomes.^[15]^ Two previous systematic reviews found significant benefits of Tai Chi exercise on sleep problems among older adults.^[16,17]^ Additionally, 2 randomized controlled trials (RCTs) demonstrated that the sleep-promoting impact of Tai Chi was noninferior to CBT, the most recommended treatment of insomnia.^[18,19]^ By contrast, Tai Chi exercise can be taught readily in either outdoor or indoor locations, and even practiced through watching professional Tai Chi videos. Moreover, Tai Chi training can be launched in a group-based pattern which may impel practitioners to stay motivated and enthusiastic to continue practicing. This is especially because of the social benefits yielded by the communications and interactions regarding Tai Chi.^[20]^ Consequently, Tai Chi exercise may be a suitable complementary and alternative therapy (CAT) to CBT to some extent as it appears to be an efficacious sleep-promoting therapy with higher cost-efficiency and convenience.

To our knowledge; however, there are limited high-quality reviews with respect to the effects of Tai Chi exercise on sleep quality among older adults. As a result, both the American and European guidelines for insomnia suggested that more evidence is warranted to judge the efficacy and safety of CAT for sleep disorders in older adults.^[10,11]^ Therefore, the current systematic review aims to evaluate the efficacy and safety of Tai Chi exercise as a CAT for sleep problems among older adults.

## 2 Methods

The protocol was registered in the international prospective register of systematic reviews database in March 2019 (CRD42019129782). This study will be executed following the guidance in the “preferred reporting items for systematic reviews and meta-analyses” (PRISMA) statement.^[21]^ Ethical approval will not be necessary since this systematic review and meta-analysis will not contain any private information of participants or violate their human rights.

### 2.1 Criteria for the included studies in the review

#### 2.1.1 Types of studies

RCTs, published in either English or Chinese that apply Tai Chi interventions on sleep problems among older adults, will be incorporated in our review. No limitations of publication status or data will be settled. Studies reported in full-text will be screened for inclusion. Additionally, those registered in the trials registries but have not been published will be contacted to ascertain whether the complete data is available.

#### 2.1.2 Types of participants

We will include trials of older adults (older than 50 years of age) who have sleep problems/complaints. We will exclude studies of people with severe comorbidities who are unable to undertake Tai Chi training.

#### 2.1.3 Types of interventions

Our systematic review and meta-analysis will be conducted based on the RCTs that solely apply Tai Chi intervention in the experimental group and placebo or other nonpharmacological therapies in the control group. Placebo or other nonpharmacological therapies may include: sleep education, waiting lists, aerobic exercise, music therapy, usual care, and so on. Tai Chi exercise can be trained in terms of Yang style, Sun style, Chen style, or a tailored form evolved from those classic Tai Chi styles. We will exclude studies of participants that undertake Tai Chi training combined with other therapies.

#### 2.1.4 Types of outcome measurements

Pittsburgh sleep quality index (PSQI) is employed as the primary outcome measurement. It differentiates “poor” from “good” sleep by measuring 7 components. These components include: subjective sleep quality, sleep duration, sleep latency, sleep disturbances, habitual sleep efficiency, use of sleep medication, and daytime dysfunction over the last month. Clients score each answer based on a 0 to 3 scale ranging from “poor” to “good” sleep quality. Specifically, the global PSQI score is 21 points, and higher scores signify severe sleep problems.^[22]^ We will use the change in the global PSQI scores from a baseline as continuous data for the meta-analysis. It should be noted that PSQI is a useful and widely translated instrument for measuring subjective sleep quality among older adults.^[23]^ PSQI is often applied in both research and medical settings due to its high cost-effectiveness, easy implementation, and high patient compliance.^[24]^

Secondary outcomes will include adverse events presented as the types and number of adverse events that are reported during trials. We may also include some other scales, such as the Athens insomnia scale and the insomnia severity index.

### 2.2 Search strategy

#### 2.2.1 Electronic searches

The following online databases will be searched from their inceptions to August 2019: PubMed, Embase, Cochrane Library, Scopus, China National Knowledge Infrastructure, and the Wanfang Database. Our search strategy is available in Appendix 1. Studies published in English and Chinese will be retrieved.

#### 2.2.2 Searching other resources.

We will screen and identify the preplanned, ongoing, and unpublished studies by searching Google Scholar, Baidu Scholar, the International Clinical Trials Registry Platform and the Chinese Clinical Trial Registry. Additionally, a manual search will be executed at the library of Duquesne University and the Nanjing University of Chinese Medicine in the event that there is any available literature missing.

### 2.3 Data collection and analysis

#### 2.3.1 Selection of studies

**Figure 1:**
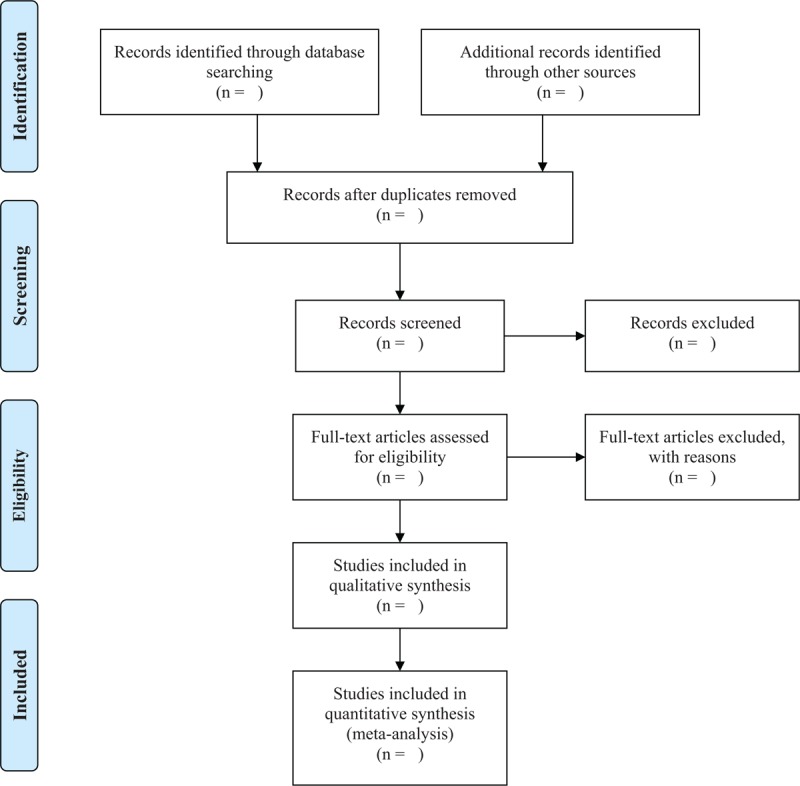
Study selection flow diagram.

Four reviewers will be divided into 2 pairs (2 in each pair: Guo and Zheng; Si and Wang), and each pair will independently screen the titles and abstracts. After removing duplicate and irrelevant articles, the 2 pairs will create 2 lists of potential studies which will be checked against each other by a supervisor (Xu) to ascertain a preliminary list. Further identification of eligible articles from the preliminary list will be completed by 2 reviewers (Wang and Guo) through applying the preplanned inclusion/exclusion criteria. A third reviewer (Ma) will make a judgment when disagreements occur. We will fill in the preferred reporting items for systematic reviews and meta-analyses (PRISMA) flowchart (Fig. 1) to demonstrate the detailed information of the study selection.

### 2.4 Data extraction and management

A Microsoft Excel spreadsheet has been created by the reviewers to record the extracted data which includes the following: the first author, publication year, sample size, trial location, age, geographic population, health status, duration and follow-up, frequency, intensity, Tai Chi style, control intervention, sleep outcome measurements, baseline and outcome data, and adverse events. This process will be completed by 2 reviewers (Si and Wang), and the results will be double-checked by a third reviewer (Ma). The corresponding author will be contacted if the extracted information is missing or unclear.

### 2.5 Risk of bias assessment

Two researchers (Si and Ma) will independently evaluate the risk of bias applying criteria provided by the Cochrane Handbook for Systematic Reviews of Interventions. This tool includes the following 7 domains:

(1) random sequence generation;
(2) allocation concealment;
(3) blinding of participants and personnel;
(4) blinding of outcome assessment;
(5) incomplete outcome data;
(6) selective reporting;
(7) other bias.

Each potential source of bias will be rated high, low, or, unclear in accordance with the extracted information in each eligible trial. Any disagreement will be determined by another reviewer (Ma).

### 2.6 Measures of the treatment effect

Standardized mean difference (SMD) will be selected over mean difference (MD) for continuous data measurement. This is due to its proved comprehensiveness in various statistical situations in comparison with unstandardized MD.^[25]^ Odds ratios (OR) will be used for dichotomous data measurement and both continuous and dichotomous data will be calculated with 95% confidence intervals (CIs).

### 2.7 Dealing with missing data

If the data of potential studies are missing, insufficient, or vague, we will attempt to contact the corresponding authors to retrieve the necessary data through email or telephone. The studies will be excluded if we cannot obtain the relevant data via the aforementioned approaches.

### 2.8 Assessment of heterogeneity

We will assess the heterogeneity applying the Chi-square and *I*^2^ test, which describes the percentage of variability in the effect estimates. *I*^2^ of 0%, 25%, 50%, and 75% signifies nil, mild, moderate, and severe heterogeneity, respectively.^[26]^ For more detailed explanation on potential heterogeneity among involved studies, we may also conduct subgroup analyses or meta-regression.

### 2.9 Assessment of publication biases

We will evaluate publication bias using the funnel plot as well as statistical tests (Egger test and Begg test).

### 2.10 Data synthesis

We will implement the meta-analyses based on at least 2 trials applying the software Stata version 12.0. SMD and OR will be applied as a means to describe the effect size for continuous and dichotomous data. We tend to select random effects for the meta-analysis model since the expected diversity of included participants and interventions may contribute to the possible existence of heterogeneity. Moreover, we will direct a narrative description if the meta-analysis is inappropriate.

### 2.11 Subgroup analysis

Subgroup analyses will be conducted which aims to explain the potential causes of heterogeneity when necessitated. The subgroup analyses will be implemented according to the physical conditions of participants (eg, good health or illness), geographic population (eg, American, Asian, or African), course of the intervention, age, or gender.

### 2.12 Sensitivity analysis

Two methods of sensitivity analysis will be employed to investigate the stability of the meta-analysis results. Specifically, the trim and fill technique will be executed as the primary means to examine our results. Moreover, if some studies have more than 1 control group, we will switch the criteria in the selection of control groups to observe whether different results will be achieved.

### 2.13 Assessment method of evidence quality

The physiotherapy evidence database (PEDro) scale will be applied to evaluate the methodological quality of the eligible studies. The PEDro scale includes 11 items, and each item has a maximum score of 10 points. A trial that is rated 6 points or more will be identified as a high-quality study. Two researchers (Si and Ma) will independently complete the process of quality evaluation. It should be noted that the use of this scale is highly recommended to assess the quality of trials for systematic reviews due to its reliability and validity.

## 3 Discussion

Tai Chi exercise may be an efficacious CAT for sleep problems, especially for older adults with moderate sleep complaints. However, both the American College of Physicians and European Sleep Research Society stated that as an approach, CAT requires further study to appraise its effectiveness in the management of insomnia.^[10,11]^ For this purpose, we directed a systematic review in 2014 to solely investigate the usefulness of Tai Chi exercise on sleep symptoms among older adults.^[17]^ Nonetheless, only 5 RCTs were involved in our previous review, which yields weak evidence. This suggests that Tai Chi might have a beneficial impact on self-rated sleep quality among healthy older adults. To further explore the efficacy of Tai Chi exercise on sleep problems, this study aims to offer more convincing and detailed information on Tai Chi for improving sleep problems in older adults. It should be noted that the present study may have potential limitations of homogeneity as a result of the various styles of Tai Chi exercise (eg, Chen style, Yang Style, and Sun style).

## Author contributions

**Conceptualization:** Yong Ma, Guihua Xu.

**Funding acquisition:** Yong Ma.

**Methodology:** Yuhao Si, Yong Ma, Guihua Xu, Cenyi Wang, Yang Guo, Jinghui Zheng.

**Supervision:** Yong Ma, Guihua Xu.

**Writing – original draft:** Yuhao Si, Cenyi Wang.

**Writing – review & editing:** Guihua Xu, Yong Ma.

## Supplementary Material

Supplemental Digital Content
