# Supplementary material for: Tai Chi exercise for sleep problems in older adults: A protocol of systematic review and meta-analysis
Source: Medicine (Baltimore). 2019 Nov 11;98(45):e17556. doi: 10.1097/MD.0000000000017556 (PMC6855661; doi:10.1097/MD.0000000000017556)
Supplement: Supplemental Digital Content [file medi-98-e17556-s001.docx]

**Appendix 1. Search strategy for Pubmed**

| No. | Search terms |
| --- | --- |
| #1 | Tai Chi [MeSH Terms] |
| #2 | Taiji [MeSH Terms] |
| #3 | Tai Chi Chuan [MeSH Terms] |
| #4 | shadowboxing [MeSH Terms] |
| #5 | taijiquan [MeSH Terms] |
| #6 | Tai Chi [Title/Abstract] |
| #7 | Taiji [Title/Abstract] |
| #8 | Tai Chi Chuan [Title/Abstract] |
| #9 | shadowboxing [Title/Abstract] |
| #10 | taijiquan [Title/Abstract] |
| #11 | #1 OR #2 OR #3 OR #4 OR #5 OR #6 OR #7 OR #8 OR #9 OR #10 |
| #12 | sleep problems [MeSH Terms] |
| #13 | sleep disorders [MeSH Terms] |
| #14 | sleep quality [MeSH Terms] |
| #15 | sleep disturbance [MeSH Terms] |
| #16 | Insomnia [MeSH Terms] |
| #17 | sleep problems [Title/Abstract] |
| #18 | sleep disorders [Title/Abstract] |
| #19 | sleep quality [Title/Abstract] |
| #20 | sleep disturbance [Title/Abstract] |
| #21 | Insomnia [Title/Abstract] |
| #22 | #12 OR #13 OR #14 OR #15 OR #16 OR #17 OR #18 OR #19 OR #20 OR #21 |
| #23 | #11 OR #22 |

The search strategy will be modified as required for other electronic databases.
